# Chemical and Biological Review of Endophytic Fungi Associated with *Morus* sp. (Moraceae) and In Silico Study of Their Antidiabetic Potential

**DOI:** 10.3390/molecules28041718

**Published:** 2023-02-10

**Authors:** Mohamed M. M. AbdelRazek, Ahmed M. Elissawy, Nada M. Mostafa, Ashaimaa Y. Moussa, Mohamed A. Elanany, Mohamed A. Elshanawany, Abdel Nasser B. Singab

**Affiliations:** 1 Department of Pharmacognosy, Faculty of Pharmacy, Badr University in Cairo (BUC), Cairo 11829, Egypt; 2 Department of Pharmacognosy, Faculty of Pharmacy, Ain Shams University, Cairo 11566, Egypt; 3 Center of Drug Discovery Research and Development, Ain Shams University, Cairo 11566, Egypt; 4 Department of Pharmaceutical Chemistry, Faculty of Pharmacy, Badr University in Cairo (BUC), Cairo 11829, Egypt

**Keywords:** antidiabetics, diabetes, *Morus*, endophytic fungi, secondary metabolites, pharmacokinetics, docking, molecular dynamics

## Abstract

The chronic nature of diabetes mellitus motivates the quest for novel agents to improve its management. The scarcity and prior uncontrolled utilization of medicinal plants have encouraged researchers to seek new sources of promising compounds. Recently, endophytes have presented as eco-friendly leading sources for bioactive metabolites. This article reviewed the endophytic fungi associated with *Morus* species and their isolated compounds, in addition to the biological activities tested on their extracts and chemical constituents. The relevant literature was collected from the years 2008–2022 from PubMed and Web of Science databases. Notably, no antidiabetic activity was reported for any of the *Morus*-associated endophytic fungal extracts or their twenty-one previously isolated compounds. This encouraged us to perform an in silico study on the previously isolated compounds to explore their possible antidiabetic potential. Furthermore, pharmacokinetic and dynamic stability studies were performed on these compounds. Upon molecular docking, Colletotrichalactone A (**14**) showed a promising antidiabetic activity due to the inhibition of the α-amylase local target and the human sodium-glucose cotransporter 2 (hSGT2) systemic target with safe pharmacokinetic features. These results provide an in silico interpretation of the possible anti-diabetic potential of *Morus* endophytic metabolites, yet further study is required.

## 1. Introduction

Type 2 diabetes mellitus (Type 2 DM) is considered one of the most prevalent metabolic disorders, affecting approximately 90% of diabetic patients [1,2]. Many medicinal plants are used in managing diabetes [3,4]. The advantage of the use of medicinal plants is due to their availability, cost-effectiveness, and higher safety [5,6,7,8]. The extensive and uncontrolled utilization of medicinal plants may add to the ecological burden in terms of overutilization of endangered and rare species and in disturbing the ecological balance [9]. In this context, endophytes associated with medicinal plants present an eco-friendly alternative source of bioactive metabolites, given that endophytes may cross talk with the host medicinal plants in terms of their biosynthetic routes or that they may be the original producers of some active ingredients, or at least may provide the host organisms with extra chemical defense to cope with the surrounding stress conditions [10,11,12,13]. The abundance of endophytic fungi within the host medicinal plants may be associated with the pharmacological actions linked to the plant part used [14]. For example, endophytic fungal metabolites associated with *Syzygium cumini* L. showed significant amylase inhibitory activity, which could be utilized in discovering new antidiabetic bioactive molecules [15]. Many fungal metabolites belonging to different classes were evaluated for their antidiabetic activities [16,17,18].

Genus *Morus* (Moraceae) comprises 17 species and 2 subspecies, distributed among temperate and tropical climates. *Morus alba*, *rubra*, and *nigra* are the most commonly known species [19]. In traditional medicine, the leaves, roots, bark, stems, and fruits of *Morus* plants are used to treat rheumatism, coughs, and inflammation. The main key bioactive chemical constituents of *Morus* genus plants have been reported as flavonoids, benzofurans, stilbenes, and Diels–Alder adducts that exhibit multiple bioactivities [20]. Moreover, *Morus* genera plants reported free radical scavenging, hypolipidemic, antioxidant, antibacterial, antiviral, and anti-inflammatory activities and were used as astringents and emollients [21]. *Morus* plants showed in vivo and in vitro antidiabetic potential with few side effects by inhibiting α-glucosidase in normal rats [22,23,24,25]. The antioxidant properties demonstrated by many plants participated at least in part in their promising bioactivities [26,27]. Metabolites such as rutin and quercetin-3-O-β-D-glucoside isolated from *M. alba* improved glucose uptake and have a positive effect on lipid accumulation in adipocytes for the management of Type 2 DM [28]. Four compounds, namely Morusalone A-D, were isolated from *M. alba* and have a mixed biosynthetic origin of polyketide, shikimic acid, and terpenoid. Their structure is close to endophytic fungi polyketides and showed potent protein tyrosine phosphatase 1B inhibitory activity (PTP1B), which is involved in the negative regulation of insulin and regulation of type 2 DM [29]. *M. nigra* revealed twelve phenolic compounds of α-glucosidase; the inhibitory activities of nigranol B and sanggenol H showed the most potent activity [28,30]. The major components of total flavonoids of *M. nigra* in in vivo study showed a reduction un prediabetes progressing to type 2 DM [31]. The antidiabetic in silico studies on *Morus* plants reported the local α-glucosidase inhibitory activities of prenylated flavone; Kuwanon C, 2-arylbenzofuran flavonoids; Moracin M and Stilbenoids; and Oxyresveratrol [32]. *Ficus* is a large important genus in the family Moraceae [33]. *Ficus religiosa* was associated with endophytic fungi that showed α-amylase enzyme inhibitory activity while the most potent fungal extract was *Cochliobolus lunatus* followed by *Abdopus aculeatus and Penicillium* sp. [34].

In the course of our interest in the research on the bioactive metabolites from endophytic fungi, we herein present a summarized review of the fungal endophytes associated with different species of *Morus* (Moraceae), focusing on the isolated 2ry metabolites and covering the period from 2008 to 2022. Since the antidiabetic role of natural products such as *M. alba* was reported mainly through local enzymatic inhibition of the α-glucosidase enzyme [35,36], no antidiabetic studies were reported on *Morus*-associated endophytes, thus encouraging us to perform in-silico molecular analysis to demarcate the activity of the previously isolated endophytic fungal metabolites associated with *Morus* species as prospective antidiabetic agents. The study was expanded to explore the antidiabetic activity through the local targets α amylase and α/β glucosidase [32,37,38,39] in addition to the systematic antidiabetic prospects of one of the emerging systemic targets for Type 2 DM, which is hSGT2, responsible for glucose reabsorption in kidneys [40,41]. The selection of hSGT2 was due to its structural similarity to several isolated compounds from *Sophora flavescens* with antidiabetic activity such as Sophoraflavanone G (A) and Kurarinone (B), which possessed IC_50_ of 4.10 and 1.70 M, respectively, on hSGT2, as well as known inhibitors Canagligfozin (C) and Empagliflozin (D) [42,43]. 


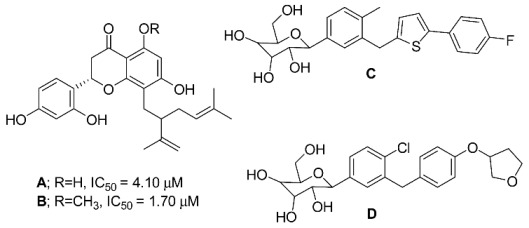


## 2. Literature Review

### 2.1. Endophytic Fungi Associated with Morus Species

**Figure 1:**
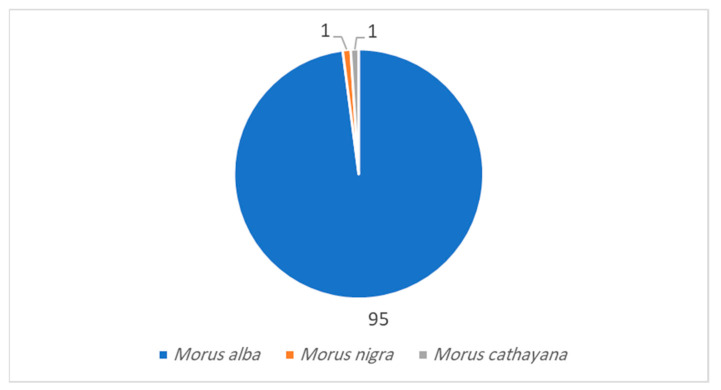
Number of identified endophytic fungi isolated from *Morus* plants.

**Figure 2:**
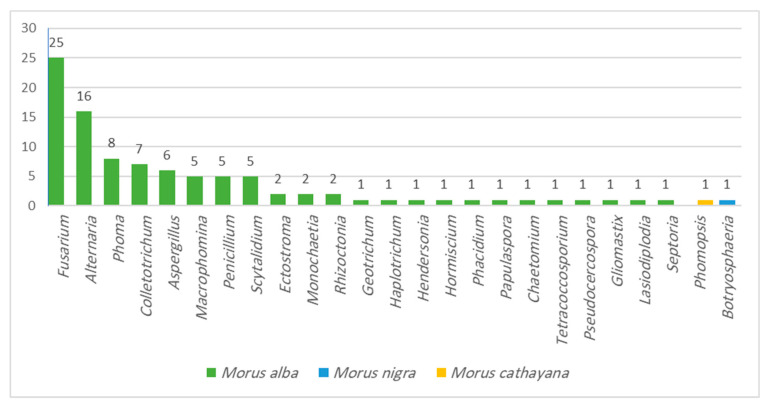
Identified endophytic fungal species from *Morus* plants.

Reviewing the literature as shown in (Figure 1 and Figure 2), 115 endophytic fungal isolates were reported from *Morus alba* leaf, stem, and root tissues; 95 (82.6%) isolates were identified, and 20 (17.4%) isolates were reported as unidentified. The most abundant identified genera of isolates reported from *M. alba* were 25 (26.3%) isolates of *Fusarium* and 16 (16.8%) isolates of *Alternaria genera* followed by a medium abundance of *Phoma* (8.4%), *Colletotrichum* (7.4%), *Aspergillus* (6.3%), and 5 (5.3%) isolates for each genus, *Macrophomina*, *Penicillium,* and *Scytalidium* [44,45,46,47,48,49,50], while one endophytic fungal strain was reported for each *Morus* genera, *Botryosphaeria* sp. for *M. nigra* [51] and *Phomopsis* sp. for *M. cathayana* [52]. However, *M. macroura* was reported to be associated with seven undefined endophytic fungal strains [19]. All endophytic fungal strains associated with *Morus* genera were reported from different locations worldwide: South Korea, China, Indonesia, Brazil, Pakistan, and the Czech Republic.

### 2.2. Chemistry of Endophytic Fungal Metabolites Associated with Morus Species

**Figure 3:**
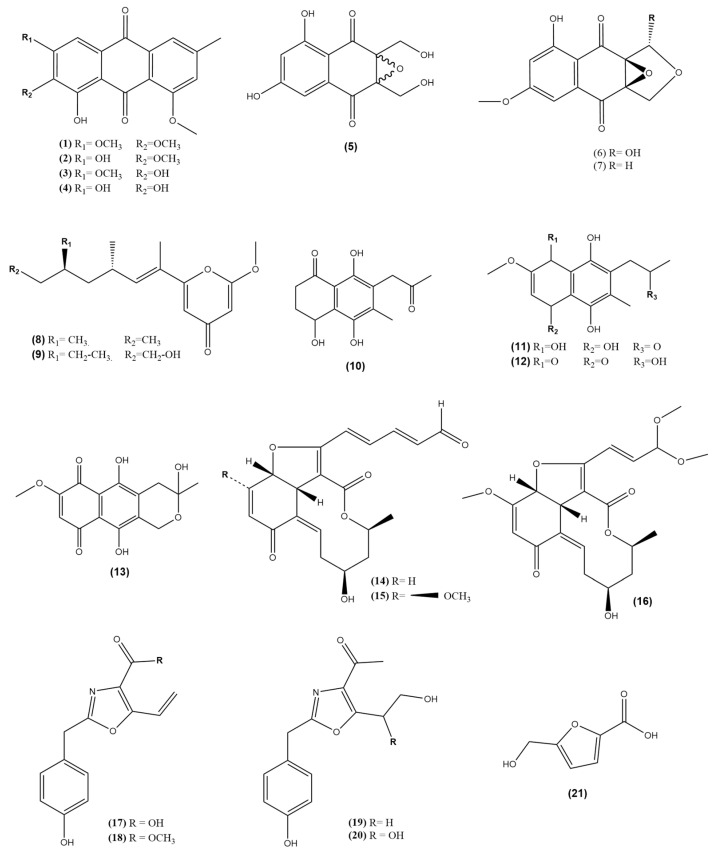
Previously isolated metabolites from endophytic fungi associated with *Morus* species.

A few works have reported the isolation of metabolites from endophytic fungi associated with *Morus* genera (Table 1 and Figure 3). A new anthraquinone, 1,3-dihydroxy-2,8-dimethoxy-6-methyl anthraquinone (**1**), was reported from the ethyl acetate extract (EtOAc) of *Colletotrichum* sp. JS-0367, isolated from the leaves of *M. alba* L. Moreover, three known anthraquinones, 1-hydroxy-2,3,8- trimethoxy-6-methyl anthraquinone (**2**), 1,2-dihydroxy-3,8- dimethoxy-6-methyl anthraquinone (**3**), Evariquinone (**4**) [44] and three quinone derivatives, epoxyquinophomopsin (**5**) and epoxyquinophomopsins A (**6**) and B (**7**), were isolated from the EtOAc extract of endophytic fungus *Phomopsis* sp. AZ1a from *M. cathayana* [52,53]. A new γ-pyrone, 6-((9‵R,11‵R, E)-13-hydroxy-9,11-dimethyloct-7-en-7-yl)-2-methoxy-4H-pyran-4-one (**8**) and a known γ-pyrone, fusarester D (**9**), were isolated from the EtOAc extract of *Fusarium Solani* JS-0169, isolated from the leaves of *M. alba* L. in addition to four known naphthoquinones: karuquinone B (**10**), javanicin (**11**), solaniol (**12**), and fusarubin (**13**) [47]. Three new colletotrichalactones, Colletotrichalactone A (**14**), Colletotrichalactone B (**15**), and Colletotrichalactone 3A (**16**), polyketides with a 5/6/10-fused ring system, were isolated from the EtOAc extract of *Fusarium Solani* JS-0169, isolated from the leaves of *M. alba* L. [46]. A new oxazole-type compound, named macrooxazole E (**17**), and macrooxazole C (**18**), macrooxazole A (**19**), and macrooxazole B (**20**), in addition to furoic acid, 5-hydroxymethyl-2-furan carboxylic acid (**21**), were isolated from the EtOAc extract of *Phoma* sp. JS0228, isolated from the leaves of *M*. *alba* L. [48]. Endophytic naphtoquinone derivatives and vanillin derivatives with benzaldehyde, 4-hydroxy-3-methoxy, or 2,5-disubstituted moieties were reported in the inhibitory activities against glucose production [54]. The unusual Colletotrichalactone polyketides with a 5/6/10-fused ring system and naphthoquinone derivative moieties may be promising targets for antidiabetic potential.

### 2.3. Biological Activities of Endophytic Fungi Associated with Morus Species

#### 2.3.1. Reported Biological Studies on Endophytic Fungal Extracts

A few studies have reported the biological evaluation of the endophytic fungal metabolites associated with *Morus* genera. The endophytic fungi crude EtOAC extracts of *Aspergillus* sp. A204, *Colletotrichum* sp. C103, and *Penicillium* sp. P306 associated with *M. alba* showed a broader antifungal spectrum [45]. *M. alba* endophytic fungi EtOAc extracts of *Phoma* sp. MJ76 and *Chaetomium* sp. showed inhibition of human immunodeficiency virus-1 (HIV-1) replication using β-galactosidase and p24 antigen in vitro assays on cell lines developed from human cervical epithelial carcinoma (TZM-bl cells) and peripheral blood mononuclear cells (PBMC) [49]. The EtOAC extract of *M. nigra* endophytic fungus *Botryosphaeria fabicerciana* (MGN23-3) showed antioxidant activity using a DPPH assay and selective antibacterial activity on gram-positive bacteria using an in vitro plate dilution method revealed by determination of minimum inhibitory concentration (MIC) and minimum bactericidal concentration (MBC) [51].

#### 2.3.2. Reported Biological Studies on Isolated Endophytic Fungal Metabolites

The isolated endophytic fungal compounds associated with *M. alba* and *M. cathayana* reported neuroprotective, antioxidant antimicrobial, antimalarial, glucose inhibitory, hemolytic, and cytotoxic activities as shown in (Table 1). Compounds (**1**), (**4**), (**11**), and (**13**) revealed protective effects using murine hippocampal HT22 cell death induced by glutamate and strongly attenuated glutamate-mediated apoptotic cell death [44,47]. Compounds (**11**), (**12**), (**13**), (**14**), (**16**), (**18**), and (**20**) showed anti-proliferative activity while compound (**16**) showed strong antioxidant power [46,48]. Compounds (**11**) and (**13**) showed antibacterial and antimalarial hemolytic activities [56]. Compounds (**18**) and (**20**) revealed antibiofilm inhibitory and destructive activities [58]. Naphthoquinones metabolites (**11**) and (**12**) presented a glucose inhibitory activity [54]. *M. cathayana* endophytic fungi, *Phomopsis* sp., revealed potential tyrosine kinase inhibitory activity of compound (**6**) [52,53].

## 3. Results and Discussion

### 3.1. Pharmacokinetic Profiling

**Figure 4:**
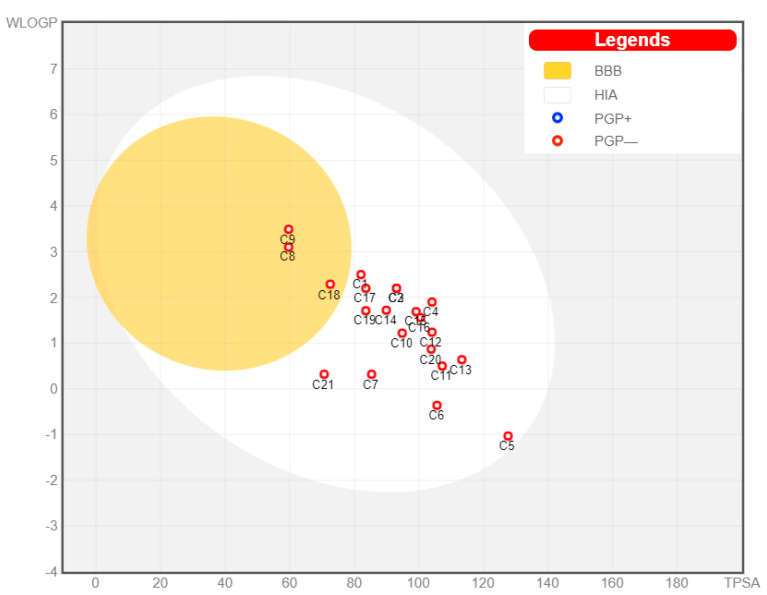
Boiled egg chart showing the predicted absorption of evaluated compounds.

As shown in (Figure 4 and Table 2), the predicted pharmacokinetic properties of the evaluated compounds revealed their high potential for gastrointestinal (GI) absorption due to their reasonable solubility. Additionally, nearly all compounds lacked permeability to the blood–brain barrier and cytochrome P2D6 (CYP2D6) inhibition, thus expanding their safety profiles, except compound (**9**). In accordance with our analysis, the high absorbability increases the potential for targeting hSGT2. Additionally, this lack of central presence adds to the benefits of these compounds through the elimination of possible side effects owing to central permeability.

### 3.2. Molecular Modelling

**Figure 5:**
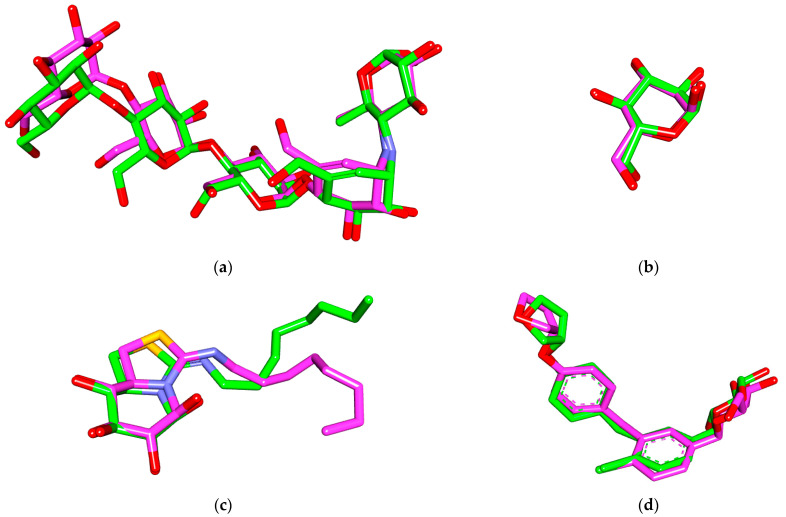
Validation results of α amylase (**a**), α glucosidase (**b**), β glucosidase (**c**), and hSGT2 (**d**) showing RMSD values of 1.22, 0.67, 1.93, and 0.47 Å, respectively. (co-crystallized pose = green, docked pose = pink).

Based on the pharmacokinetic results, the antidiabetic potential of the compounds was investigated locally and systematically through screening of action against α amylase, α/β glucosidase enzymes, and human sodium-glucose cotransporter 2 (hSGT2). Autodock Vina successfully performed the docking process of the select compounds in three targets, α amylase (PDBID: 2QV4), β glucosidase enzymes (PDBID: 2XWE), and hSGT2 (PDBID: 7VSI) for screening of their potential in antidiabetic therapy while MOE08 was used for α glucosidase (PDBID: 3A4A) after unsuccessful attempts using Vina. The docking protocol was validated through docking of the co-crystallized ligand in each receptor, followed by comparing the co-crystallized pose and docked pose and calculation of RMSD between them. As shown in (Figure 5), α amylase with co-crystallized acarbose showed an RMSD of 1.22 Å, while α and β glucosidase RMSD was 0.67 and 1.95 Å, respectively. Similarly, empagliflozin, which is co-crystallized in hSGT2, had an RMSD of 1.01 Å.

All compounds demonstrated favorable binding to the three selected targets as evidenced by the obtained negative values of docking scores in kcal/mol in (Table 3). For comparative analysis of the antidiabetic abilities of the evaluated compounds, acarbose was used as a reference for inhibitory activity on α amylase and β of glucosidase, while empagliflozin was used as an hSGT2 reference inhibitor.

#### 3.2.1. α Amylase Interaction

**Figure 6:**
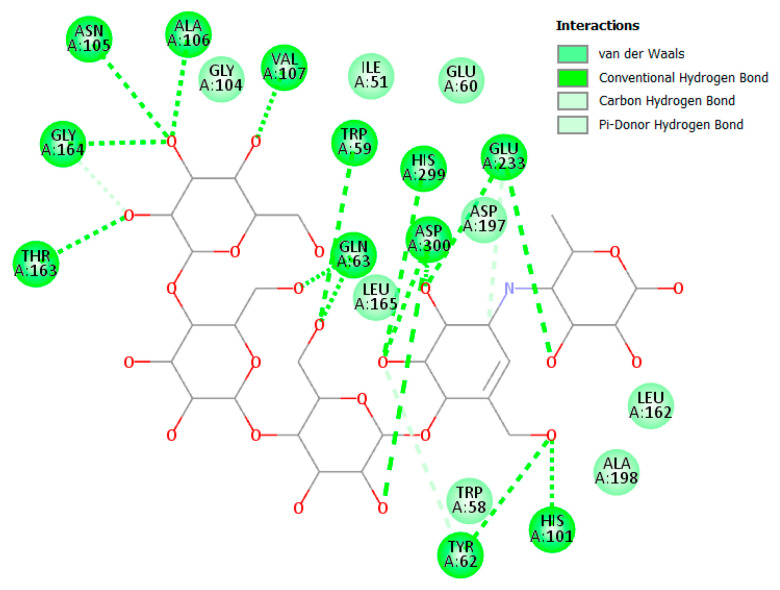
Two-dimensional binding interaction of acarbose with α amylase.

**Figure 7:**
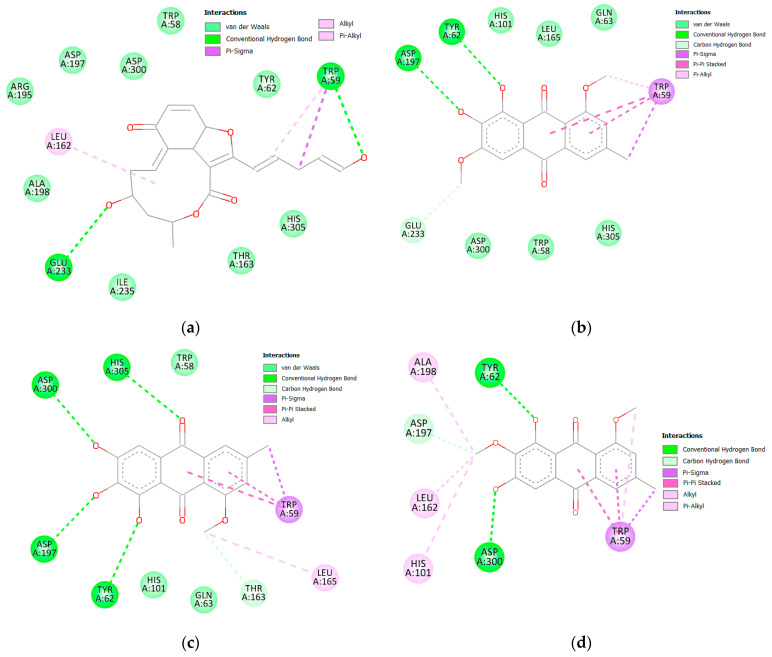
Two-dimensional interactions of α amylase with compounds **14** (**a**), **3** (**b**), **4** (**c**), and **2** (**d**).

**Figure 8:**
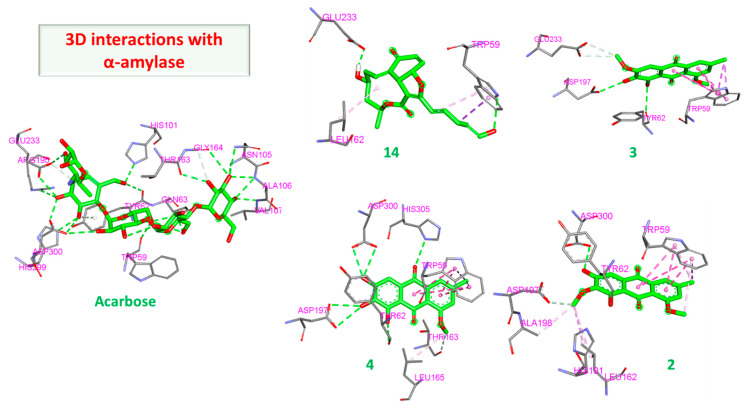
Three-dimensional interactions of α amylase with acarbose and top-scoring compounds (**14**, **3**, **4** and **2**).

Both acarbose and tested compounds demonstrated negative bind scores hinting at the favorable interactions with the enzyme. Acarbose showed the highest affinity with a score of −9.70 kcal/mole, while (**14**), (**3**), (**4**), and (**2**) demonstrated the highest affinities of −8.80, −8.50, −8.50, and −8.40 kcal/mol, respectively. Upon inspection of 2D interactions, it becomes clear that the hydrophilic nature of acarbose enables it to form multiple hydrogen bonds with several α amylase residues such as Trp59, Tyr62, Gln63, His101, Asn105, Ala106, Val107 Thr163, Arg195, Glu233, and Asp300 (Figure 6). Although the compounds were able to interact with common amino acids such as Trp59, Tyr62, Thr163, Glu233, and Asp300, they were less able due to the more lipophilic characteristics of the compounds. However, the top-scoring compounds compensated for this deficiency through the formation of hydrogen bonds with other amino acids in the binding site, namely, Leu162, Leu165, Asp197, Asp198, and Asp305 (Figure 7 and Figure 8).

#### 3.2.2. α and β Glucosidase Infarction

**Figure 9:**
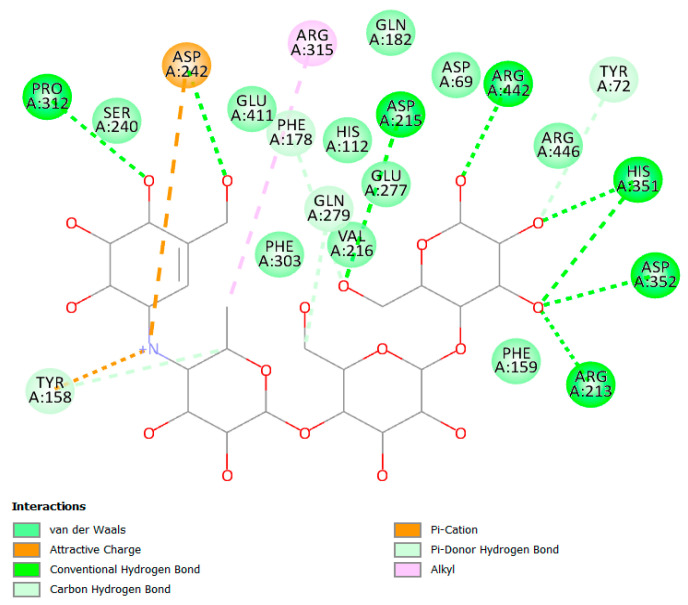
Two-dimensional binding interaction of acarbose with α glucosidase.

**Figure 10:**
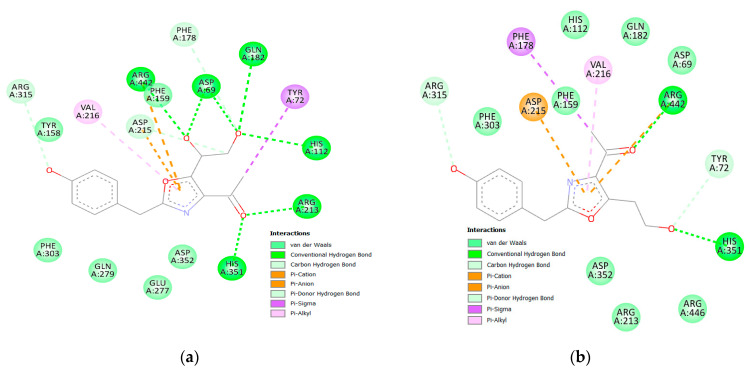
Two-dimensional interactions of α glucosidase with compounds **20** (**a**) and **19** (**b**).

**Figure 11:**
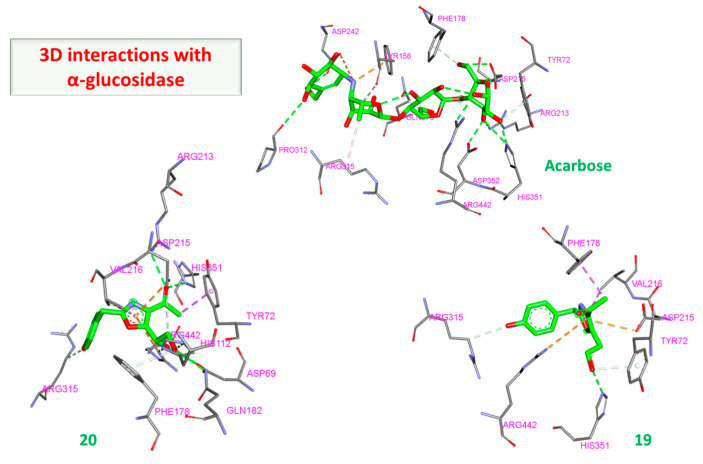
Three-dimensional interactions of α-glucosidase with acarbose and top-scoring compounds (**20** and **19**).

α/β glucosidases contributed to the treatment of type 2 DM by breaking down the glycosidic by the α isoform and the aryl and alkyl glycosides, disaccharides, and small oligosaccharides by the β isoform [32,59]. The binding of acarbose to the α and β isoforms was −8.97 and −8.70 kcal/mol, respectively. Although the compounds showed favorable binding in both cases, the binding was stronger in the β isoform in nearly all instances suggesting a partial preference for β rather than α. The hydrophilic nature of acarbose enables it to form multiple hydrogen bonds with several α glucosidase residues such as Tyr72, Tyr158, Phe178, Arg213, Asp215, Asp242, Gln279, Pro312, His351, Asp352, and Arg442 (Figure 8). Additionally, ionic interactions were observed as well with Tyr158 and Asp242 in addition to one hydrophobic bond with Arg315 (Figure 9). Among the tested compounds, only (**20**) and (**19**) were the ones with the closest scores of −6.96 and −6.58 kcal/mole, respectively. Although (**20**) maintained two similar ionic interactions with Asp215 and Arg462, its less hydrophilic nature only accommodated the formation of a lower number of hydrogen bonds than acarbose, which explains its lower score. This observation becomes more evident upon inspection of the interactions of (**19**), which has fewer hydrophilic groups capable of the formation of hydrogen bonding (Figure 10 and Figure 11).

**Figure 12:**
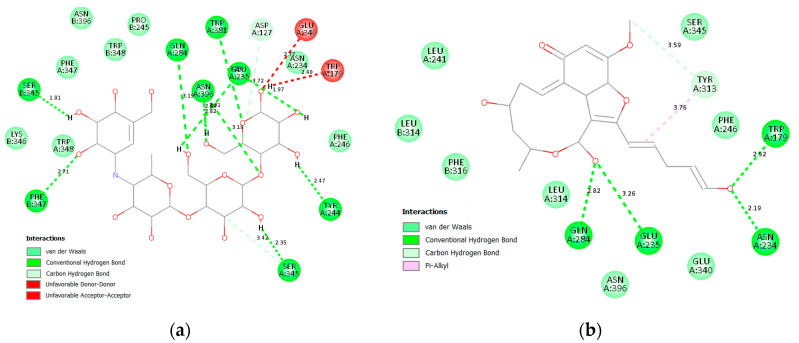
Two-dimensional interactions of β glucosidase with acarbose (**a**) and compound **15** (**b**).

On the other hand, the docking results against β glucosidase were more intriguing. (**15**) marginally outperformed acarbose with scores of −9.10 and −8.70 kcal/mol, respectively. Additionally, compounds (**14**) and (**16**) attained nearly similar scores, achieving −8.60 and −8.50 kcal/mol, respectively. A closer inspection of the interactions explains why (**15**) achieved this score. Upon closer inspection, it binds more tightly to β glucosidase, the distance of the hydrogen bonds formed is optimal, ranging from 2.19 to 3.59 Å, and the hydrophobic bond was 3.76 Å with Tyr313. In contrast, acarbose formed hydrogen bonds ranging from 2.71 Å to 4.14 Å. Additionally, the binding of acarbose creates unfavorable binding and steric tension with Trp179 and Glu340 (Figure 12). The combined effects of these two factors rationalize the marginal superiority of (**15**) over acarbose.

**Figure 13:**
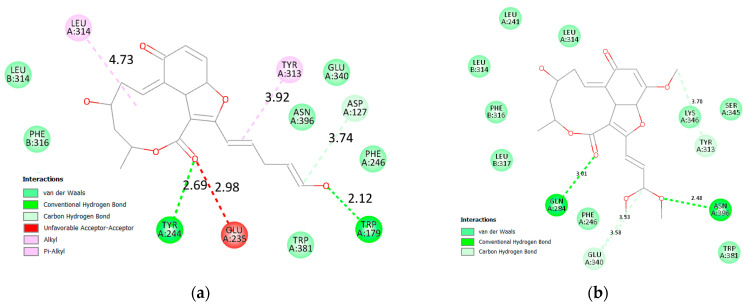
Two-dimensional interactions of β glucosidase with compounds **14** (**a**) and **16** (**b**).

**Figure 14:**
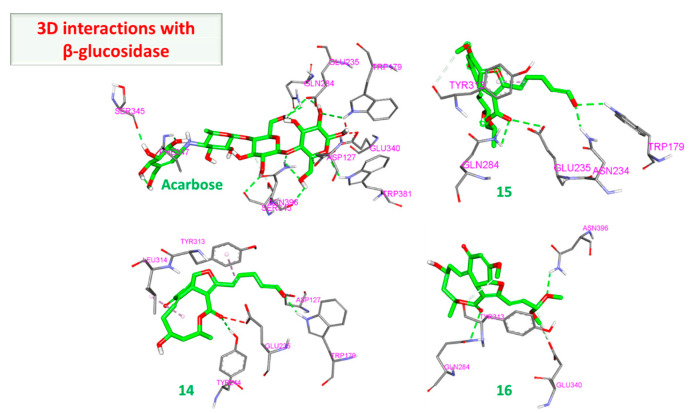
Three-dimensional interactions of β-glucosidase with acarbose and top-scoring compounds (**15**, **14**, and **16**).

The impact of this unfavorable binding and hydrogen bond distance becomes more evident when viewing interactions of compounds (**14**) and (**16**) (Figure 13 and Figure 14). In the case of (**14**), despite the short distance hydrogen bonds, there is unfavorable interaction with Glu235. On the other hand, there are no unfavorable interactions with (**16**), but the hydrogen bond distances are longer.

#### 3.2.3. hSGT2 Interaction

**Figure 15:**
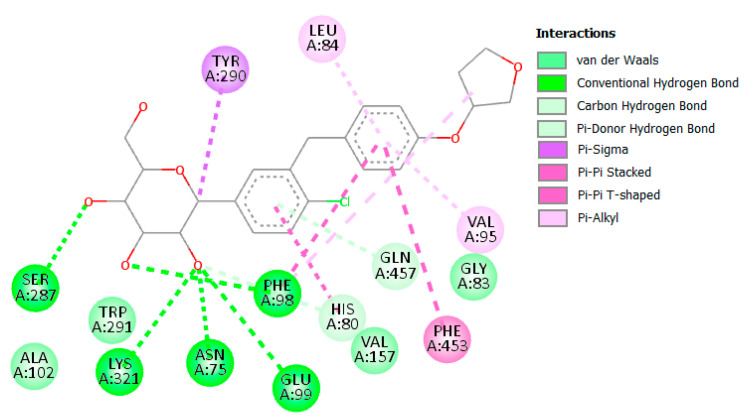
Two-dimensional interactions of hSGT2 with empagliflozin.

Glucose reabsorption via the kidney is one of the contributing factors in type 2 DM, and as such, targeting this process is an intriguing prospect in antidiabetic therapy [60]. Human sodium-glucose co-transporter proteins are responsible for this machination and as such hSGT2 (PDBID: 7VSI) was selected, which also contained co-crystallized empagliflozin and was used for validation and comparison [61]. As shown in (Figure 15), the sugar moiety of empagliflozin is involved in many hydrogen bond interactions with Asn75, Phe98, Glu99, Ser287, and Lys321. Additionally, several hydrophobic interactions were also observed with His80, Leu84, Val95, Phe98, Tyr290, and Phe453. This plethora of interactions resulted in empagliflozin scoring −11.60 kcal/mol.

**Figure 16:**
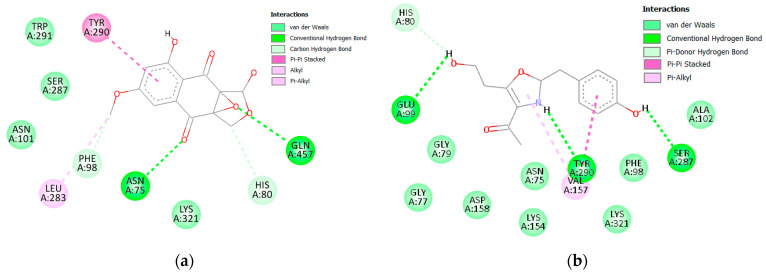
Two-dimensional interactions of hSGT2 with compounds **6** (**a**) and **19** (**b**).

**Figure 17:**
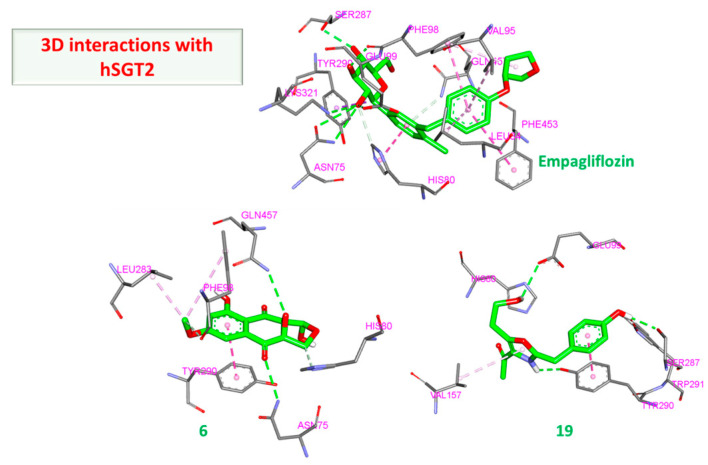
Three-dimensional interactions of hSGT2 with empagliflozin and top-scoring compounds (**6** and **19**).

Although no compound was able to outperform empagliflozin, the closest binding was observed with (**6**) and (**19**), both scoring −8.80 kcal/mol. Several members scored −8.70 kcal/mole (**7**, **8**) and (**20**), and only (**14**) scored −8.50 kcal/mol. The 2D interactions of both (**6**) and (**19**) reveal their interactions with His80 and Tyr290 (Figure 16 and Figure 17). Individually, (**6**) interacted with certain five amino acids as empagliflozin (Asn75, His80, Phe98, Tyr290, and Gln457) in addition to Leu283 while (**19**) interacted with only four similar amino acids (His80, Glu99, Ser287, and Tyr290) and Val157.

### 3.3. Molecular Dynamic Simulations and Generalized MMGBSA Calculations

Extensive investigation of the binding modalities and stability under realistic physiological settings was performed using molecular dynamic simulations. The proteins were simulated for 50 ns with and without the compounds using the Schrodinger Maestro package. The root mean square deviation (RMSD) of the protein–ligand complexes was calculated to ascertain the stability of the binding interactions, while the root mean square deviation (RMSD) of the ligands was used to assess the conformational changes the ligands undergo over the estimated simulation time period. Additionally, the root mean fluctuation (RMSF) of the amino acid residues and their contact with ligands was computed.

**Figure 18:**
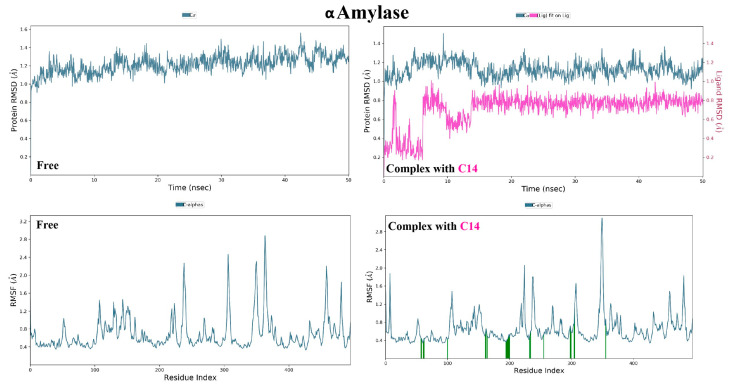
Molecular dynamic simulation results of α glucosidase in absence and presence of compound **14**. ((**Top**): RMSD plots of protein, protein-ligand, and ligand, (**Bottom**): RMSF plots of protein residue).

Analysis of the free amylase’s trajectory reveals a relatively uniform behavior, as demonstrated by the nearly plateaued RMSD value of 1.40 Å (Figure 18). On the other hand, the effect of binding of compound (**14**) is observed as a consistent decrease in RMSD, indicating restriction of enzymatic movement and binding stability. Similarly, the same conclusion can be drawn when comparing the RMSF values of amino acid residues in the presence and absence of (**14**) and finding that fluctuations are restricted. In addition, (**14**) demonstrated conformational uniformity throughout the entire procedure with an RMSD of 0.80 Å.

**Figure 19:**
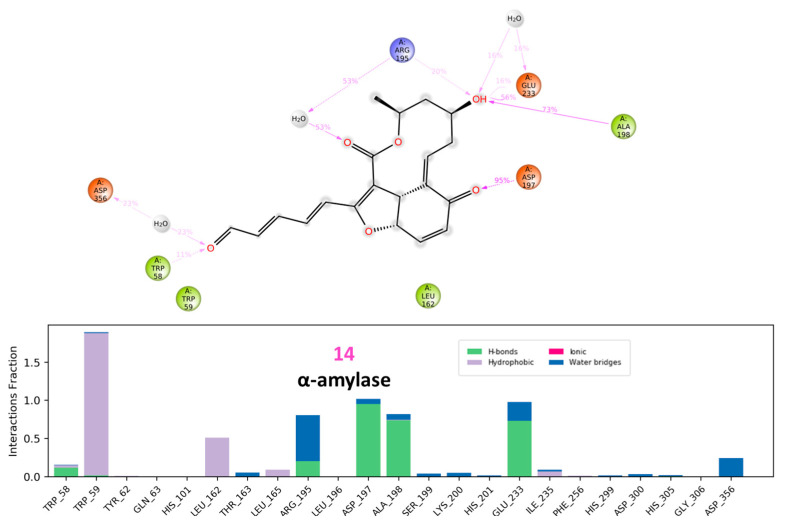
Ligand-protein contacts of α glucosidase in presence of compound **14**.

As shown in Figure 19, α-amylase trajectory analysis revealed the interaction of (**14**) with Trp59 and Glu233 continuously in addition to the appearance of several other interactions with Asp197 and Ala198.

**Figure 20:**
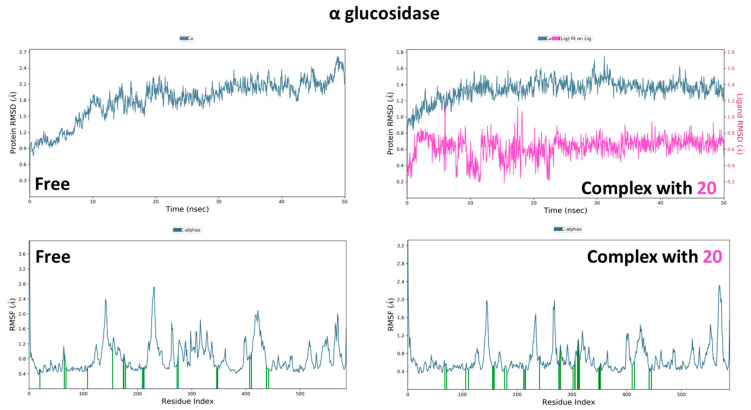
Molecular dynamic simulation results of α glucosidase in absence and presence of compound **20**. ((**Top**): RMSD plots of protein, protein-ligand, and ligand, (**Bottom**): RMSF plots of protein residue).

**Figure 21:**
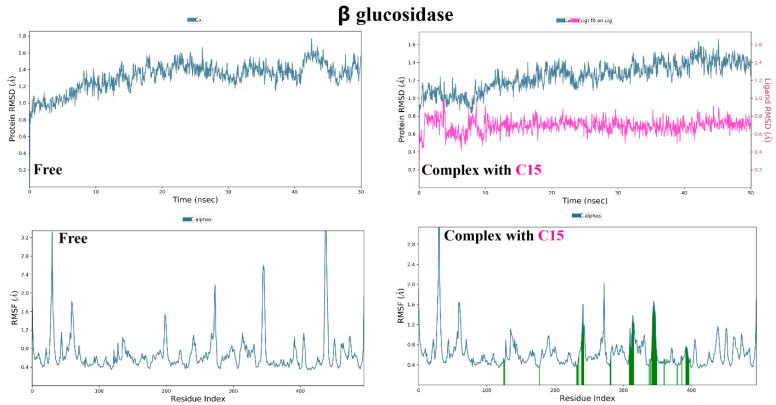
Molecular dynamic simulation results of β glucosidase in absence and presence of compound **15**. ((**Top**): RMSD plots of protein, protein-ligand, and ligand, (**Bottom**): RMSF plots of protein residues).

Trajectory analysis of the free α and β glucosidase shows relative homogeneity in behavior as demonstrated by its near plateau of RMSD at around 1.40 and 1.45 Å, respectively (Figure 20 and Figure 21). On the other hand, the effect of binding of compounds (**20**) and (**15**) is observed as a consistent decrease in RMSD, implying the restriction of enzymatic movement and the stability of binding. Similarly, the same observation can be drawn when examining the RMSF values of the amino acid residues in the presence and absence of compounds, in which residues show limitation in fluctuations when (**20**) and (**15**) were present. Finally, both (**20**) and (**15**) exhibited conformational uniformity throughout the process as well with RMSD values of 0.75 and 0.70 Å, respectively.

**Figure 22:**
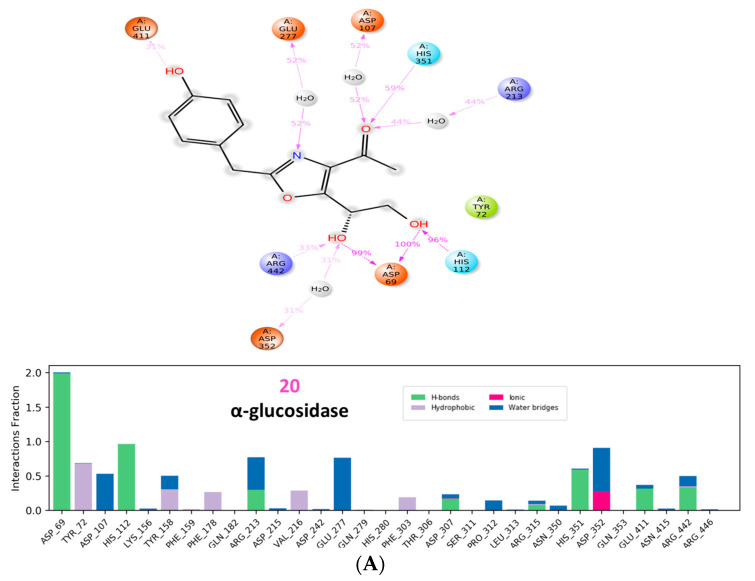

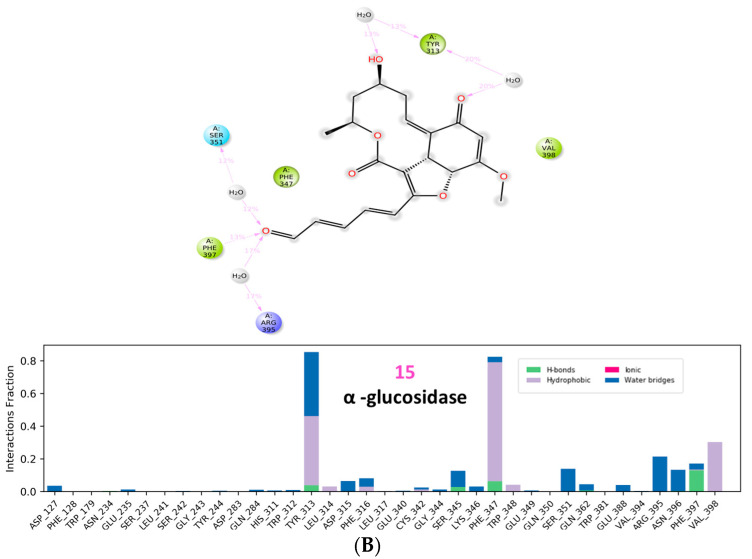
(**A**) Ligand-protein contacts of α glucosidase in presence of compound **20**. (**B**) Ligand-protein contacts of β glucosidase in presence of compound **15**.

Analysis of the various interactions of (**20**) across the whole simulation duration (Figure 22) illustrated the consistency with previous docking, in that the two vicinal hydroxyl groups were involved with Asp69 in addition to His112 and Arg442 throughout the simulation. On the other hand, several hydrophobic interactions of (**15**) were revealed with Tyr313 and Phe347.

**Figure 23:**
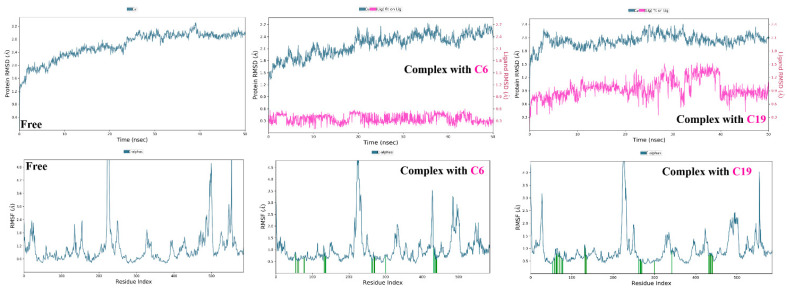
Molecular dynamic simulation results of hSGT2 in absence and presence of compounds **6** and **19**. (Top: RMSD plots of protein, protein-ligand, and ligand, Bottom: RMSF plots of protein residues).

Similarly, analysis of molecular dynamic simulation of the hSGT2 without any ligand demonstrated a plateau RMSD around 3 Å, while both (**6**) and (**19**) reduced RMSD to 2.40 and 2.10 Å, respectively. Their binding was also reflected in RMSF values as shown in (Figure 23). Finally, both compounds (**6**) and (**19**) exhibited conformational uniformity throughout the process as well with RMSD values of 0.50 and 0.90 Å, respectively.

**Figure 24:**
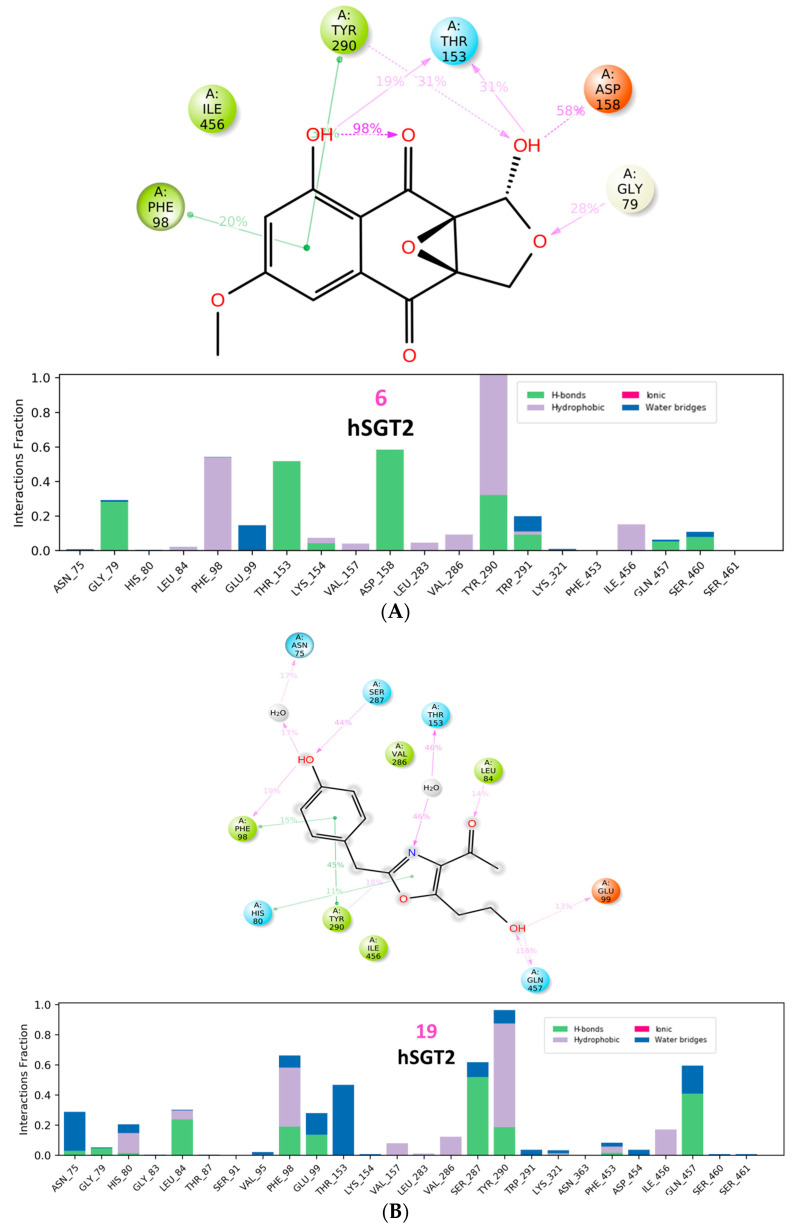
(**A**) Ligand-protein contacts of hSGT in presence of compound **6**. (**B**) Ligand-protein contacts of hSGT in presence of compound **19**.

Interactions of (**6**) and (**19**) were also analyzed throughout the simulation interval (Figure 24); interactions with Phe98 and Tyr290 were the most frequent in both cases. However, individually, the hydrophilic nature of (**6**) enabled the formation of hydrogen bonds Thr153 and Asp158.

Another tool for assessing the stability under solvated conditions as in physiological systems is the calculation of binding free energy. Among these tools, Molecular Mechanics Generalized-Born Surface Area (MM-GBSA) is one of the most frequently used methods deployed. The difference in solvent (water) interaction energy with the free receptor, free ligand, and complex is used to calculate the GB and SA energy terms. The molecular mechanics energy obtained from the interaction between the receptor and the ligand under the considered force field is used to compute MM [62]. The lower the predicted binding free energy of a ligand–protein complex, the more stable the complex will be and the greater the ligand’s activity and potency (Table 4). For all simulations, the complexes maintained close energy scores at the beginning and end. This consistency of the binding energies of the targets to different compounds hints at stable binding throughout the simulation.

## 4. Material and Methods

### 4.1. Eligibility Criteria for the Review

Studies were selected according to the isolated bioactive compounds from endophytic fungi associated with *Morus* species from 2008 to July 2022 and the biological activities conducted on these compounds during this period. The search spanned several databases such as PubMed and Web of Science.

### 4.2. Pharmacokinetic Profiling

The ADME profile provided by the SwissADME website (www.swissadme.ch; accessed on 6 September 2022) is an excellent web-based tool for the prediction of pharmacokinetic parameters [63,64]. Compounds were imported and predicted as demonstrated by the previous literature [65].

### 4.3. Molecular Docking

The selected targets were obtained from a protein data bank (PDBID: 2QV4, 3A4A, 2XWE, and 7VSI) [35,66,67,68,69]. The compounds and proteins were prepared and converted to the appropriate format using Open Babel and PyRx [70,71]. The docking was performed using Autock Vina software (1.2.0) and MOE08 [72,73]. Root mean square deviation (RMSD) was calculated and the interactions were viewed using Biovia Discovery Visualizer 2021 [74,75,76,77,78].

### 4.4. Molecular Dynamic Simulations and Generalized MMGBSA Calculations

The Schrodinger Desmond package was utilized for simulations of molecular dynamics utilizing the “OPLS4” forcefield for 50 ns, as detailed in previous studies [79]. The solvation was performed using “TIP3P” water molecules using an “Octadecahedron” solvation box. The binding free energy of the examined protein–ligand complexes was computed using the MM-GBSA method, which integrated molecular mechanics (MM) force fields with a Generalized Born and Surface Area continuum solvation solvent model using the Schrödinger Prime software [80,81].

## 5. Conclusions

The chronic nature of diabetes mellitus and its crippling effects on the quality of life drives the research for the identification of new agents to improve antidiabetic management. Traditional medicine provides an enormous source of medicinal plants and phytochemicals with established use. However, the environmental burden of using these plants increases the importance of finding alternative sources of bioactive molecules from eco-friendly endophytic fungi. Taking advantage of the antidiabetic effects of *Morus* plants, this study sought to explore the *Morus* endophytic fungal metabolites responsible for this property. The previous literature revealed a total of twenty-one compounds under this criterion. The pharmacokinetic properties of the compounds were calculated to narrow down the potential targets and ascertain their safety. The compounds showed safe properties with high intestinal absorption, low blood–brain barrier permeability, and no interactions with cytochrome P2D6. Expanding on these data, we evaluated the compounds’ antidiabetic properties through their capability to affect local and systemic targets in the form of α/β glucosidase and human sodium-glucose cotransporter 2 (hSGT2), respectively. The compounds showed promising potential against all targets with varying degrees in terms of binding scores as well as the stability of such interactions. One of the most promising agents is Colletotrichalactone A (**14**); it inhibited α amylase and both isoforms of glucosidase with a greater preference for β than α. Moreover, it was among the top-scoring agents that inhibited hSGT2. This highlights its potential in antidiabetic management locally and systematically. Another candidate is Colletotrichalactone B (**15**) which outperformed acarbose inhibition on β glucosidase. The result of our study provides an in silico interpretation of the antidiabetic potential of *Morus* endophytic metabolites as well as providing sufficient evidence for future research on these agents and linking their pharmacological actions to the host, assuming that endophytic fungi are a more eco-friendly leading source of promising bioactive compounds than plant sources.

## Figures and Tables

**Table 1:** Reported metabolites isolated from endophytes associated with *Morus* species.

No	Compound Class/Name	M.W.	Reported Biological Assay	Biological Activity	Source	References
	**Anthraquinone**					
**1**	1-hydroxy-2,3,8- trimethoxy-6-methyl anthraquinone	328.32	ROS (EC_20_) NO (EC_50_) PGE2 (EC_20_) Neuroprotective (HT22 Viability%)	100 µM >100 µM >100 µM 75% at conc. 12 µM	*Colletotrichum* sp. JS-0367 associated with *M. alba* Leaves (South Korea)	[44,55]
**2**	1,3-dihydroxy-2,8-dimethoxy-6-methyl anthraquinone	314.29	ROS (EC_20_) NO (EC_50_) PGE2 (EC_20_) Neuroprotective (HT22 Viability%)	51.1 µM 27 µM 49.5 µM 42% at conc. 12 µM		
**3**	1,2-dihydroxy-3,8- dimethoxy-6-methyl anthraquinone	314.29	ROS (EC_20_) NO (EC_50_) PGE2 (EC_20_) Neuroprotective (HT22 Viability%)	>100 µM >100 µM 75.3 µM 30% at conc. 12 µM		
**4**	Evariquinone	300.27	ROS (EC_20_) NO (EC_50_) PGE2 (EC_20_) Neuroprotective (HT22 Viability%) Antioxidant DPPH (IC_50_)	71.2 µM >100 µM >100 µM 50% at conc. 12 µM 42.2 μM		
	**Quinone**					
**5**	Epoxyquinophomopsin	266.21	-	-	*Phomopsis* sp. AZ1a associated with *M. cathayana* Twigs (Indonesia)	[52,53]
**6**	Epoxyquinophomopsin A	278.22	TK inhibition (%)	16–20%		
**7**	Epoxyquinophomopsin B	262.22	TK inhibition (%)	19–20%		
	**Pyrone**					
**8**	6-((9‵R,11‵R, E)-13-hydroxy-9,11-dimethyloct-7-en-7-yl)-2-methoxy-4H-pyran-4-one	280.36	Hepatoprotective (HT22 Viability%)	41% at conc. 12.5 µM	*Fusarium Solani* JS-0169 associated with *M. alba* leaves (South Korea)	[47,54,56]
**9**	Fusarester D	294.39	Neuroprotective (HT22 Viability%)	<1% at conc. 12.5 µM		
	**Naphthoquinones**					
**10**	Karuquinone B	264.28	Neuroprotective (HT22 Viability%)	<1% at conc. 12.5 µM		
**11**	Javanicin	294.30	Neuroprotective (HT22 Viability%) Glucose production inhibition (IC_50_) Antimicrobial Activity Antimalarial activity Hemolytic Activity Cytotoxicity (IC_50_)	50% at conc. 12.5 µM 3.8 µM 25 μg/mL (*S. aureus*, *P. aeroginosa*, *S. epidermidis*, *E. coli*) 50 μg/mL (*K. pneumoniae*) 290 µM IC_50_ = 1389 µM (14% at 200 µM) 37.1 µM (MCF7) >100 µM (DU145) 23.1 µM (HeLa) >100 µM (A549) 39.1 µM (B16F10) 13 µM (MDA-MB321) 3.3 µM (H4IIE-C3)		
**12**	Solaniol	292.29	Neuroprotective (HT22 Viability%) Glucose production inhibition (IC_50_) Cytotoxicity (IC_50_)	<1% at conc. 12.5 µM 4.4 µM 9.5 µM (H4IIE-C3)		
**13**	Fusarubin	306.27	Neuroprotective (HT22 Viability%) Antimicrobial Activity Antimalarial activity Hemolytic Activity DPPH (IC_50_) Cytotoxicity (IC_50_)	90% at conc. 12.5 µM 1.56 μg/mL (*S. aureus, E. coli, P. aeruginosa*), 3.125 μg/mL (*S. epidermidis*), 12.5 μg/mL (*K. pneumoniae*) IC50 = 176 µM IC_50_ = 1914 µM (11.3% at 200 µM) 60 µM 7.7 µM (MCF7) 4.2 µM (DU145) 15.6 µM (HeLa) 10.3 µM (A549) 1.5 µM (B16F10) 16 µM (MDA-MB321)		
	**Polyketides**					
**14**	Colletotrichalactone A	356.37	Cytotoxicity (IC_50_)	35 µM (MCF7)	*Colletotrichum* sp. JS-0361 associated with *M. alba* leaves (South Korea)	[46]
**15**	Colletotrichalactone B	386.40	Cytotoxicity (IC_50_)	>100 µM (MCF7)		
**16**	Colletotrichalactone 3A	406.43	Cytotoxicity (IC_50_)	25 µM (MCF7)		
	**Oxazole**					
**17**	Macrooxazole E	245.23	Cytotoxicity (IC_50_)	No activity on (MCF7) and (LNCaP)	*Phoma* sp. JS0228 associated with *M. alba* leaves (South Korea)	[48,57,58]
**18**	Macrooxazole C	259.26	Biofilm inhibitory% Biofilm destructive% Cytotoxicity (IC_50_)	59% (125 μg/mL) against *S. aureus* 48% (125 μg/mL) against *S. aureus* 29 µM (MCF7), 36 µM (LNCaP)		
**19**	Macrooxazole A	261.28	Biofilm inhibitory% Biofilm destructive%	No activity against *S. aureus* No activity against *S. aureus*		
**20**	Macrooxazole B	277.28	Biofilm inhibitory% Biofilm destructive%	43% (125 μg/mL) against *S. aureus* 31% (125 μg/mL) against *S. aureus*		
	**Furoic acid derivative**					
**21**	5-hydroxymethyl-2-furan carboxylic acid	142.03	-	-		

(M.W.) = Molecular weight; (TK) = tyrosine kinase inhibition; (ROS) = reactive oxygen species; (NO) = nitric oxide production; (PGE2) = prostaglandin E2; (DPPH) = 2,2-diphenylpicrylhydrazyl for free anti radical scavenger antioxidant activity; (IC_50_) = half-maximal inhibitory concentration; (EC_20_) = concentration of compound that produces 20% biological effect; (EC_50_) = concentration of compound that produces 50% biological effect; (HT22) = murine hippocampal cell line; (MCF7) = human breast cancer cell line; (LNCaP) = prostate cancer cell line; (HeLa) = Human cervical cancer cells; (DU145) = Human prostate cancer cells; (A549) = Adenocarcinomic Human alveolar basal epithelial cells; (MDA-MB321) = Human breast cancer cells; (B16F10) = Mouse Skin melanoma cells; (H4IIE-C3) = Rat hepatoma cell line.

**Table 2:** Predicted ADME profiles for the compounds using SWISSADME.

Compound	TPSA	Log P	Solubility	GI Absorption	BBB Permeability	CYP2D6 Inhibition
**1**	82.06	2.57	Moderately	High	No	No
**2**	93.06	2.22	Moderately	High	No	No
**3**	93.06	2.22	Moderately	High	No	No
**4**	104.06	1.86	Moderately	High	No	No
**5**	127.59	−0.3	Soluble	High	No	No
**6**	105.59	0.25	Soluble	High	No	No
**7**	85.36	0.77	Soluble	High	No	No
**8**	59.67	3	Moderately	High	Yes	No
**9**	59.67	3.32	Moderately	High	Yes	Yes
**10**	94.83	1.22	Soluble	High	No	No
**11**	107.22	0.66	Soluble	High	No	No
**12**	104.06	1.53	Soluble	High	No	No
**13**	113.29	0.92	Soluble	High	No	No
**14**	89.9	1.73	Soluble	High	No	No
**15**	99.13	1.59	Soluble	High	No	No
**16**	100.52	1.44	Soluble	High	No	No
**17**	83.56	1.97	Soluble	High	No	No
**18**	72.56	2.36	Moderately	High	Yes	No
**19**	83.56	1.67	Moderately	High	No	No
**20**	103.79	0.91	Soluble	High	No	No
**21**	70.67	0.16	Soluble	High	No	No

(TPSA) = Total Polar Surface Area; Log P = Consensus Log P; (GI) = Gastrointestinal; (BBB) = Blood Brain Barrier; (CYP2D6) = Cytochrome P2D6.

**Table 3:** Docking results of select compounds against α amylase, α/β glucosidase, and hSGT2 in kcal/mol.

Compound	α-Amylase	α Glucosidase	β Glucosidase	hSGT2
**1**	−8.10	−4.52	−7.60	−6.10
**2**	−8.40	−4.44	−7.80	−6.20
**3**	−8.50	−3.72	−7.60	−6.80
**4**	−8.50	−3.91	−8.00	−7.90
**5**	−6.90	−6.06	−6.50	−7.80
**6**	−7.30	−5.73	−7.30	−8.80
**7**	−7.10	−5.65	−7.30	−8.30
**8**	−6.90	−5.79	−7.20	−8.30
**9**	−7.00	−6.04	−7.30	−7.70
**10**	−7.20	−5.69	−7.00	−8.30
**11**	−7.10	−5.65	−7.30	−7.90
**12**	−7.40	−4.42	−7.30	−7.50
**13**	−8.00	−5.52	−7.70	−8.10
**14**	−8.80	−3.34	−8.60	−8.50
**15**	−8.00	−3.38	−9.10	−6.60
**16**	−7.70	−2.57	−8.50	−7.50
**17**	−6.90	−5.79	−7.40	−8.70
**18**	−6.70	−6.05	−7.50	−8.70
**19**	−7.10	−6.58	−7.30	−8.80
**20**	−7.10	−6.96	−7.40	−8.70
**21**	−5.10	−5.46	−5.40	−6.00
Acarbose	−9.70	−8.97	−8.70	----
Empagliflozin	----	----	----	−11.60

**Table 4:** Docking results of select compounds against α amylase, α/β glucosidase, and hSGT2 in kcal/mol.

Complex	Compound		dG Binding	dG Binding Coulomb	dG Binding (NS)	dG Binding (NS) Coulomb
α-amylase	**14**	Start	−63.40	−36.48	−64.65	−36.54
		End	−67.56	−30.09	−69.59	−30.46
α glucosidase	**20**	Start	−38.54	−22.09	−42.82	−25.58
		End	−36.46	−17.85	−41.39	−23.17
β glucosidase	**15**	Start	−31.37	−9.02	−32.49	−9.22
		End	−38.47	−9.47	−39.29	−9.41
hSGT2	**6**	Start	−61.81	−22.73	−64.53	−24.44
		End	−50.556	−8.38	−51.09	−8.81
	**19**	Start	−51.94	−22.66	−53.70	−21.56
		End	−47.63	−16.95	−50.73	−17.53

## Data Availability

Data are reported in the article or are available from the corresponding authors upon reasonable request.
